# Finding knowledge: how youth identify their candidacy and sources of information regarding sexual and reproductive health in rural KwaZulu-Natal. South Africa

**DOI:** 10.1186/s12889-025-22595-6

**Published:** 2025-04-16

**Authors:** Ntombizonke A. Gumede, Siphesihle Hlongwane, Vuyiswa Nxumalo, Dumile Gumede, Maryam Shahmanesh, Janet Seeley, Guy Harling

**Affiliations:** 1 Africa Health Research Institute, KwaZulu-Natal, South Africa; 2 Durban University of Technology, Durban, South Africa; 3 Institute for Global Health, University College London, Mortimer Market Centre, London, WC1E 6JB UK; 4 Department of Global Health and Development, London School of Hygiene and Tropical Medicine, London, UK; 5 School of Nursing & Public Health, College of Health Sciences, University of KwaZulu- Natal, Durban, South Africa; 6 MRC/Wits Rural Public Health and Health Transitions Research Unit (Agincourt), School of Public Health, University of the Witwatersrand, Johannesburg, South Africa

**Keywords:** Health communication, Sexual and reproductive health, Candidacy framework, Youth, South Africa

## Abstract

**Background:**

Despite efforts by government, non-governmental organizations, local communities and families, sexual and reproductive health (SRH) behavior and outcomes among adolescents and young adults (“youth”) remain of concern in South Africa. Improving outcomes requires better understanding of how youth navigate and engage with potential sources of SRH information, so interventions can align with the contextual landscape.

**Methods:**

We conducted secondary analysis of qualitative data, including interviews and group discussions, from five studies conducted in uMkhanyakude district, KwaZulu-Natal. We adapted the existing Candidacy Framework from its original focus on service use to apply to communication. We re-coded the transcripts using a thematic coding framework.

**Results:**

Youth identified their candidacy for SRH information when they could not understand what others were saying about sexual health, when they feared illness in themselves or significant others, and when experiencing a health issue. Participants expected different types of information from healthcare providers, family members and peers, and had a nuanced understanding of the strengths and weaknesses of each source. Stigma related to youths’ SRH and their precarious socioeconomic circumstances limited channels for communication and the ability for shared interpersonal knowledge to impact health behavior.

**Conclusions:**

Identification of candidacy for SRH information in this setting was largely ad-hoc, but once aware of need, youth were able to identify and discriminate between multiple information sources. However, this information landscape was strongly shaped by formal provider and parent views of what youth should know and do, and peer sources limited own knowledge. Future interventions could address concerns at various points on the candidacy journey.

## Background

Young South Africans aged 10–24 (hereafter “youth”), particularly adolescent girls and young women (AGYW), experience high burdens of HIV [1, 2], other sexually transmitted infections (STIs) [3, 4] and early pregnancy [5]. Improved health interventions targeting this age group are thus an imperative. Efficacious prevention modalities exist for all the above sexual and reproductive health (SRH) concerns, beginning with barrier contraceptives. For HIV, other biomedical prevention technologies include medical male circumcision, antiretroviral therapy (ART) as prevention for people living with HIV, and Pre-Exposure Prophylaxis (PrEP) for uninfected individuals [6, 7]. More generally, youth are exposed to much SRH-related information, including about HIV and gender-based violence (GBV), and nominally have broad access to SRH services [8]. However, despite numerous efforts, use of protective SRH modalities remains low nationally [9].

This low level of uptake reflects potential barriers for youth in relation to SRH at multiple levels, including the interpersonal, familial, community, service-provider and societal. At the societal level, economic precarity can limit youths’ options regarding SRH, for example by promoting transactional relationships [10]. Community social norms regarding acceptable behavior can also play an important role in decisions about sexual behavior and protection [11, 12]. Despite initiatives intended to create a conducive environment for SRH services among youth [13], adolescents seeking SRH services at health facilities often face disapproval and reprimands from providers [14, 15], limiting actual service access [16–18]. Interpersonal barriers exist around the use of some contraceptive methods, such as condoms, which is often limited by power imbalances in sexual decision making, expectations of trust and perceived decreased pleasure [19–22].

In addition to external factors, youth face internal barriers to enacting preventative SRH behaviors. These include making sense of SRH-related meanings, sexual philosophies and decisions, in the context of rapid cognitive and socio-emotional development [23]. Despite abundant SRH resources, in practice youth will only take preventative measures if they are aware of opportunities, see themselves as candidates for safer methods, and are able to comfortably access and implement them [24, 25]. These steps are relevant both for technologies (e.g., PrEP, condoms) and behaviors (e.g., sexual and alcohol abstinence, monogamy). At each step careful communication of both SRH risks and opportunities for positive health outcomes, provided by trusted others, can help overcome such gaps.

Health communication has long been acknowledged as potentially helpful in the development of positive sexual attitudes and behaviors among youth [26]. Communication is integral to many South African public health strategies; government funds SRH mass communication programs – including media campaigns, advertisements, magazines and TV drama series – with the aim of improving transmission knowledge, risk perceptions, social norms and behaviors [27]. Communication has been central to campaigns which have contributed to declines in HIV infections, teenage pregnancy and GBV [28, 29].

Parent-child discussions have been shown to positively influence SRH behavior choices [30–32]. However, in practice such conversations are often unidirectional, fear-driven, vague and fraught with discomfort [33]. Such discussions can be improved by parental skills and confidence building initiatives such the Collaborative HIV/AIDS and Adolescent Mental Health Programme (CHAMP) intervention [34], which work in part by reducing taboos around SRH conversations and raising parents’ SRH knowledge [35].

Although parents are frequently identified as the preferred source of SRH information [30, 36], they are not the only source of sexual socialization: siblings, extended family members, peers, educators, school nurses, books and the internet are also used [31, 33, 35, 37]. Alongside access to sexual content on television and the internet, youth can obtain information at health education sites [32, 38].

Within South Africa, KwaZulu-Natal province has both the highest HIV prevalence [1] and the lowest rate of modern contraceptive use [9]. However, there is limited evidence on the facilitators and barriers to SRH communication among youth in the province, particularly in rural areas. We therefore sought to understand what information rural youth in KwaZulu-Natal possess about SRH, where they get information from and what challenges they face in accessing a wider range of SRH information. Through critically examining youths’ daily lived experience and context we aimed to better understand how this influences their decisions, and thus identify challenges for health communication at different levels and how these challenges might be overcome.

### Theoretical framework

For youth to absorb information and access services, they must first recognize that sources of SRH information are intended for them and then see themselves as intended recipients for SRH services. This process of becoming candidates has been defined as “how people assess their eligibility for accessing health services and how they legitimize their interaction and engagement with services” [39]. This Candidacy Framework centrally recognizes that eligibility for any intervention is a social construct. Accordingly, it emphasizes the multiplicity, diversity and complexity of “candidacy” and examines how candidacy is shaped by people’s past interactions (with health services, healthcare professionals, interventions, access and information) and their socio-economic and cultural contexts. The framework provides an infrastructure for understanding how youth reflect on their experiences within the cultural and institutional discourse in which they are embedded, and how this influences their ultimate behaviors [24, 25, 40, 41].

We adapted the original framework developed by Dixon-Woods and colleagues [40], which focuses on healthcare service uptake, to apply it to SRH communication (Table 1). We focus on how youth choose who to approach for information about SRH, how they start conversations on the topic and how they process the communication they receive. Using a candidacy lens also helps us consider how pre-existing resources and relationships affect SRH decisions and subsequent behaviors. Such decisions are shaped by institutions such as kinship, family, peer relations and religious and spiritual structures, and articulated and enforced through interpersonal interactions [42]. Notably, societal ideas about femininity and masculinity, and mores, norms and sanctions relating to SRH behaviors are structured by individuals’ social and economic situations to determine how they perceive the relevance and accessibility of SRH interventions in their lives [43].

**Table 1:** Candidacy framework

Stage	Original description	Application for SRH communication in youth
Identification of candidacy	How do individuals come to view themselves as legitimate candidates for particular services	How do youth come to see themselves as having an unmet need for SRH information?
Navigation of services	Knowing how to make contact with appropriate services in relation to identified candidacy	How do youth identify who might be able and willing to provide the information they need?
Permeability of services	Includes the level of explicit and implicit gatekeeping within a service and the complexity of its referral systems; in addition, it refers the ‘cultural alignment’ between users and services	How do they reach the right person(s), to what extent do these individuals limit their openness regarding SRH to youth?
Appearing at services and asserting candidacy	The work that individuals must do to assert their candidacy in an interaction with a healthcare professional	How do youth start conversations, or how to others start conversations with youth about SRH?
Adjudications by professionals	Candidacy as expressed by service-users is validated or otherwise by healthcare professionals and this influence subsequent offers of services	How do others decide which type of information to communicate to which youth, at which times?
Offers of/resistance to services	Emphasizes that follow-up services may be appropriately or inappropriately offered and that these may or may not be acted upon by service-users	How do youth react to the information that they are provided; in what ways is the offered information accepted or rejected?
Operating conditions and local production of candidacy	This incorporates factors that influence decisions about subsequent service provision (e.g. the resources available for addressing candidacy) and the kinds of contingent relationships that develop between professionals and service-users over a number of encounters	How do longstanding social relationships affect how youth approach others, and how, and what, others are willing to discuss with youth.

Source: Adapted from [41]

## Methods

### Study context and setting

We conducted a secondary analysis of qualitative data gathered across five studies (Table 2) conducted within the geographic boundaries of the Africa Health Research Institute’s Population Intervention Platform (AHRI PIP) site in the Hlabisa subdistrict of uMkhanyakude district in KwaZulu-Natal (KZN) province, South Africa. This largely rural area with one town, KwaMsane, covers ~ 845 km^2^ with ~ 140,000 individuals in about 20,000 households [44].

**Table 2:** Summary of primary studies used in this analysis

Study	Aim	Inclusion criteria	Location	Methods
Perceptions (Ngwenya et al., 2018 [45])	To identify structural components and aspects of a community, including the culture and environment which contribute to HIV and TB infection to support development of targeted infection prevention interventions.	Adults aged ≥ 19	Two rural or and two semi-rural sites	Spiral walks; community observations; focus-group discussions (*n* = 22); key informant interviews (*n* = 14); in-depth interviews (*n* = 25)
Mobility (Bernays et al., 2020 [46])	To explore youths’ perceptions, experience and exposures associated with migration, and how this shapes HIV-risk behaviors.	Youth aged 16–24	One peri-urban and one rural site	Longitudinal in-depth interviews (*n* = 48)
Hope (Desmond et al., 2019 [48])	To explore adolescents’ perceptions of their local community, their place within it, their substance use and sexual behavior, and how they understand hope and happiness in these contexts	Youth aged 15–17	One rural site	Key informant interviews (*n* = 30); focus group discussions (*n* = 4)
m-Africa (Adeagbo et al., 2019 [24])	To understand ways in which an mHealth intervention could be developed to overcome barriers to existing HIV testing and care services and promote HIV self-testing and linkage to prevention and care in a poor, HIV hyperendemic community in rural KwaZulu-Natal, South Africa	Adults aged ≥ 18	Two rural communities	In-depth interviews (*n* = 54); focus group discussions (*n* = 9)
DREAMS (Zuma et al., 2019 [49])	To understand complex social interactions and culturally informed norms which influence perceptions, experiences and how the changing HIV landscape is navigated by adolescents and young adults	Adolescents and adults aged ≥ 10	Throughout the area	Focus-group discussions (*n* = 10); longitudinal in-depth interviews (*n* = 58)

### Participant selection and sampling strategy

The five studies selected were all conducted in the AHRI PIP area between 2017 and 2021 using qualitative methods. Each study used a different sampling strategy, although all were purposive based on the aims of each study; the aims and inclusion criteria for each study are indicated in Table 2. Three were stand-alone research projects: “Perceptions” focused on community perceptions of local boundaries, and of HIV vulnerability, prevalence and risk [45]; “Mobility” compared the experiences of those who had recently moved from rural settings to town with those who had not moved away, and how (non)mobility influenced their HIV risk, preventive and treatment seeking behavior [46, 47]; “Hope” explored adolescents’ perceptions of their local community, how they fitted within it, their substance use and sexual behavior, and how they understood hope and happiness in these contexts [48]. The other two studies were nested within broader projects: “m-Africa” was the qualitative process evaluation of a project to design online care pathways and mobile diagnostics for population HIV testing, prevention and care in community settings [24] and “DREAMS” was a longitudinal study, of which the qualitative aspect explored how a wide range of social and clinical interventions were perceived, experienced and could hinder or facilitate HIV treatment and prevention [49]. The short form of each title is used hereafter to identify studies and quotations from them.

### Data collection and analysis

Within each study participants were asked questions about social context, social interactions and sexual health using unstructured or semi-structured interview guides (details on all study instruments provided in the primary analysis papers cited in Table 2). Data had previously been collected and then either transcribed and translated from isiZulu to English (where necessary) or summarized in detail by the field teams and checked by each study’s investigators. For the purposes of this paper we re-analyzed and re-coded transcripts in both English and isiZulu using NVIVO 12, revisiting recordings where necessary. After reading one interview from each study, we developed a thematic coding framework informed by this study’s objectives, so that emerging findings could inform the framework. This framework was used for coding with constant comparison across the team to ensure consistency.

We took an interpretative approach, based on the concept that our everyday knowledge is acquired through shared meanings, perceptions, and the use of language which are all social constructs. We therefore began by describing how participants understood the context within which they live their lives as a framework for understanding their decisions and behaviors. We then considered how study participants perceived their eligibility for access to SRH information considering the first four components of the adapted Candidacy Framework. Our focus was on how youth gained access to SRH services, so we did not include the last two stages of the framework relating to communication content (how others decide what to say, and how recipients react to what they are told) in this paper.

### Ethics

This research was performed in accordance with the Declaration of Helsinki. All five underlying studies were previously approved by research ethics committees (REC) at University of the KwaZulu-Natal (UKZN) or Human Sciences Research Council in South Africa and the London School of Hygiene and Tropical Medicine or University of Southampton in the United Kingdom (depending on the study). This analysis was approved by UKZN’s Biomedical REC and University College London’s REC as falling within the scope of the original studies. Both the original studies and this reanalysis were reviewed and approved by AHRI’s Community Advisory Board. All participants in the original studies provided written informed consent; where children under 18 years of age were interviewed, written informed consent from parents or guardians was also obtained.

## Results

### The social context of sexual and reproductive health

Communication about SRH in this setting occurs in the context of widespread social, economic and environmental vulnerabilities. While participants reported that the drivers of SRH risk varied by age, sex and wealth, wider vulnerabilities directly and indirectly affected SRH decisions. Youth was consistently described as an inherently risky time in life, grounded in exploration, however these risks are exacerbated by social and economic structures. Limited educational and employment opportunities for youth were perceived to lead to early school dropout. Once not in education, employment or training, youth spent most of their time with peers, making peer-pressure a key SRH determinant. Material insecurity was widely acknowledged to contribute to unintended pregnancy, sexual violence and acquisition of HIV and other STIs. Lack of clean water, good quality housing and healthy food were all mentioned as reasons for increased vulnerability to HIV, in part through an inability to access condoms and to negotiate safer sex. Economic imbalances were seen as leading to power imbalances, which in turn placed young women and men at risk of negative SRH outcomes:

*We used to visit to their stores so that (laughing)*,* so that they will talk to us and then get something like money or goods. You know by the time a person is giving you something you too have to give him something [sex] in return (female*,* 22yrs*,* FGD*,* Perceptions)*.

*By that time you no longer care about your life*,* because you had been through a lot of difficulties in life*,* so having this person as part of your life is better because she has money*,* so you will sleep with her without a condom. (young male*,* no age given*,* FGD*,* Perceptions)*

Alcohol affected reasoning around sex, with substance misuse also named as a common coping mechanism for boredom and stress. The interaction of poverty and alcohol was often seen as driving sexual behavior, with alcohol sometimes given as an excuse for risky behavior, even when economic motives were present:

*In our days girls will do anything for money*,* so people in ezipotini (taverns*,* bars*,* places of alcohol) will buy [alcohol]. So*,* you find that someone will buy alcohol and spend their money in order to sleep with a girl. Thus*,* when someone who is HIV positive does that*,* no one questions that because everyone is intoxicated*,* reasoning is skewed. I think people can do anything for money and forget about the risks involved*,* and that they can be exposed to things like that. More especially*,* when one has been drinking*,* sexual activity tends to be a highly likely occurrence. (male*,* 23*,* IDI*,* Mobility)*

### Identification of candidacy

At the first stage of candidacy, participants viewed health communication as something both to guide them in healthy sexual relations and decisions, and to make them aware of the potential negative consequences of sex – notably HIV, STIs and pregnancies. Recognition of a need for SRH information arose from at least four sources. First, some identified themselves as candidates for some form of SRH information due to fear of non-specific infection:

*What encourages me as I have said is that I’m scared of getting any disease*,* that’s what encourages me to go to the programs so then I know how to protect myself from certain things that are sex related (female*,* 18*,* IDI*,* DREAMS)*.

Second, some became aware that they lacked SRH information after hearing others discuss SRH-related topics that they did not understand, which prompted them to seek clarification:

*Sometimes at the clinic I would see both males and females*,* but the males would say that “ubhajiwe” [Literally meaning stuck*,* but also Gonorrhea*,* also referred to as ‘drop’] and I would not understand why they would say that they are stuck. they are stuck*,* where are they stuck? what is happening*,* you know. Eventually*,* I did find (what it meant) because I talk a lot with the boys at school. So*,* one time in the classroom during an LO [life orientation] period I asked the teacher about what does “ukubhajwa” mean*,* and they then told me what it is. I then noted every time that when a person says they were stuck that they meant that they had drop. (female*,* 22*,* IDI*,* DREAMS)*

Third, some participants were motivated by witnessing a close relative, friend or acquaintance being ill:

*…. like my older brother*,* the one who died*,* he first contracted TB and was taking his medication*,* he then stopped taking his treatment to continue drinking alcohol. Then after some time*,* he contracted HIV you see*,* he was not taking pills*,* he was just throwing it in the toilet. That is when I saw that HIV is actually killing people*,* I now take an HIV test every month… (male*,* 25*,* IDI*,* m-Africa).*

Finally, participants experienced health issues themselves, so SRH-related information followed a health event rather than preceding it. A common example of this was menarche, which was often described as confusing, frightening, distressing and awkward due to an absence of past sex education:

*I was scared…I thought it was something bad and I was scared to tell my parents.…(In the end) I told them*,* I gained courage to tell them…I don’t know but I felt embarrassed.…She [Granny] is the person I stay with and talk to about everything.…. I first told her [mom] and she advised me then I told granny… [Mom] told me that menstruating is not a bad thing*,* if I have not engaged in sexual intercourse. I should naturally get my periods*,* so I shouldn’t be afraid*,* I must tell granny. I told [granny] that I am menstruating*,* and she didn’t have a problem. She told me what to do. (Female*,* 16*,* IDI*,* DREAMS)*

### Navigation, permeability and assertion of communication desire

Once youth had considered themselves candidates for SRH information, the process of finding the right sources and asking for help was complex. Participants reported widespread stigma regarding youth sexuality, which limited opportunities for open communication with parents, partners or health service providers – which in turn resulted in gaps in contraceptive knowledge and use. Nevertheless, youth were able to identify several sources of SRH information – including healthcare providers, schools and teachers, family members and peers – and deliberately sought different types of information and clarification from each. Same-gender conversations were seen as important, especially for functional communication on sexual matters, due to shared experiences and common understandings:

*Let me put it like this*,* for me it depends on what kind of sickness you have*,* whether it will be a female or a male…For me as I said I tell my father if there something I’m experiencing. Perhaps I will make a call since my father stays at work*,* ‘father I have something like this and that in my body’*,* since he is a male he will readily tell me that it caused by this. Or I tell my brother ‘My brother I have such and such thing’*,* since he is older than me maybe he has experienced it. Then if I see that they cannot help me I then tell my mother*,* just go straight to her*,* mother I’m like this…Since even the females cannot just rush to a male about something in her body*,* rather she rushes to the sister or the mother. If she sees that they cannot help her she can then move on to ask others (male*,* 22*,* IDI*,* DREAMS).*

#### School and clinic

Healthcare providers – both at clinics and in schools – are common providers of SRH information. They are specifically expected to provide factual information, especially on biological aspects which are not typically covered at home. Clinics are associated with HIV prevention interventions (e.g., condoms, circumcision) and were thus seen as a good source for biomedical information. School-based sex education is also perceived to provide factual biological information, including broad Life Orientation classes and specific programs delivered by organizations such as Isikhondlakhondla, which focused on tuberculosis and HIV prevention, and Mpilonhle which focused on SRH services and support. Although HIV is widely discussed in the community, sex education classes provided greater depth of information on issues such as HIV, STIs and PrEP:

*What we have learnt at school is that HIV is not transmitted through hugging a person*,* but you can acquire HIV if you share someone’s important things like a toothbrush and things of some sort because it might happen sometimes that a person is affected and has bleeding gums and someone will use the same toothbrush and HIV can be transmitted through mother to child but it is better now that we have treatment and to use a condom if you are engaging in sexual intercourse with someone with HIV (female*,* 15*,* IDI*,* DREAMS).*

Information received in such classes also dispelled fears acquired from other sources that limited uptake of preventative measures:

*People from [a local NGO] came to our school and explained about circumcision and told us to go and register for it and when they told us there were forms which we were supposed to fill before being circumcised. At the time*,* I was still afraid of doing it and they explained that it is okay if you are still scared you can always do it next time at the local clinic*,* and I waited until they came back again and that is when I went to the clinic to do it.….Here at home they used to tell me that this thing (circumcision) kills*,* and my uncles told me that they too are alive without being circumcised. [Nevertheless] We saw those who returned*,* and we went there after them (male*,* 17*,* IDI*,* DREAMS).*

However, sex education messages can be misinterpreted:

*According to what I learnt in Life Orientation; a condom kills your baby.…Your babies are left in a condom and after using it*,* you dump it right?…Yes*,* you are dumping your baby and that is why I am saying PrEP is better (female*,* 17*,* IDI*,* DREAMS)*.

Despite the perceived benefits of clinic- and school-based information, there were barriers to gaining information from these sources. These included travel distance and waiting times for clinics, and the limited ability of youth to actively engage with staff and teachers who lacked a willingness to engage in conversations rather than transmit information.

#### Family

Participants talk about SRH with a wide range of family members, but there are at least three subsets of relatives who provide information in different ways. Siblings, cousins and other similar-aged individuals are a common first point of communication. Conversations with older family members tended to be didactic, rudimentary and focus on instilling fear and cautioning about negative consequences, without focusing on the emotional aspects of being involved in an intimate relationship:

*Yes*,* we do talk with grandma…She tells us that if we sleep with a boy and when she takes us to the virginity testing and find out that we have had sex*,* she will kill us. (female*,* 10*,* IDI*,* DREAMS)*

Finally, parents are a mixed source of advice. While parent-child relationships can allow SRH discussions, taboos and traditions can make direct discussion difficult, and often youth are not able to initiate discussion:

*In the olden days parents would have a person who will lead and educate young girls in a household*,* which means it was difficult to get HIV. (female*,* 17*,* IDI*,* DREAMS)*

*Yes*,* there can be a reason for speaking to your mother*,* and there is a reason for you to speak with your father*,* because there are things you cannot discuss with your mother… let me make an example*,* if maybe a maiden has started seeing someone? Perhaps a partner wants to do right by her*,* she would go to her sister first before her mother. Then if their sister says no*,* since sisters can also have fear then they can say go and talk to mother… You cannot just go to tell your father about such things at least you can tell your Aunt. (male*,* 22*,* IDI*,* DREAMS*)

*My mother’s older sister (initiates the conversation)..No*,* I haven’t (started the conversation) [Why not? ]…Nothing*,* there is nothing that makes me want to talk to her about it…I am afraid of her since she is my elder….But I wouldn’t go to her and talk about it. (female*,* 18*,* IDI*,* DREAMS).*

For some participants, parents readily provided information based on their past experiences with the aim of preventing their children from going through the similar experiences.

*Yes*,* she (mom) sat me down and spoke to me…. she was advising me. She said I’m old enough since I’m already having my periods*,* and when we reach the adolescence stage*,* we sometimes end up doing things we should not be doing. She said a lot of things*,* and she also told me she doesn’t want me to end up like her*,* being HIV positive was not intentional but it was my father’s fault. I asked if dad infected her when she was pregnant or not*,* she said no*,* luckily I was born by then. (female*,* 17*,* IDI*,* DREAMS)*

*I do get it from my parents…(from)…my mother and sisters…She usually say if you do this there will be the consequences and you will end up here.[Interviewer: When you do things like what? ] Like sex*,* you will end up with diseases*,* and being pregnant…I accept it (information). Because I know*,* and I see others. Some end up with diseases and pregnant. People don’t hide their statuses anymore*,* they talk about it. (female*,* 18*,* IDI*,* DREAMS)*

There was also a concern that parents have given up trying to communicate about SRH with youth, due to the perception that youth did not listen even when their parents tried to be understanding, friendly and openly communicate.

*[A]fter they have fallen pregnant they (parents) would say is that what we said to you?… you find that a parent would try to be friendly to a child and say she must speak with her about anything*,* but the child end up disrespecting a parent to the extent that they would sneak through windows at home. (female*,* 17*,* IDI*,* DREAMS)*

#### Peers

Peer influence on SRH decision making was widely recognized and seen as potentially positive in broadening knowledge. Youth recognized friends as important discussants for topics relating to feelings, emotions, dating and relationships. Peers also provided an outlet for discussion of SRH issues youth were uncomfortable discussing with authority figures, in part because similar ages and experiences made sharing easier and friends were accessible. For example, conversations with friends arose after health education visits:

*Okay in fact because this [conversations with friends] usually happens because they [people from the clinic] were visiting to educate us. They normally talk about things and you find that one becomes shy to ask from those who give awareness. But afterwards you find that someone asks you*,* also that you may like to know if sexual transmitted diseases are similar to HIV*,* if it is curable. (female*,* 18*,* IDI*,* DREAMS).*

Peers provided an opportunity to have a discourse, minimizing the risk of stigma or a sense of threat, fear or discomfort:

*I normally talk with my friends and my elder brothers who are of course not my age…I think it is because I feel comfortable speaking with them and to speak whatever I want to speak in an open manner. Sometimes I ask them as joke but yet knowing that the question is serious. (male*,* 19*,* IDI*,* DREAMS)*

Despite friends often being seen as a potential source of information, there was also concern that they could not always be trusted not to deliberately give the wrong information or share secrets:

*No*,* I talk to… or to find that you talk to your friend*,* it’s indeed not easy here because it would be better if there’s someone like you (AHRI) as you’re working here whom you can share your story with. Because you’ll try to share it with your friend and they would just make fun of you; make it a joke. Like when you find out that you’ve suffered from drop*,* then he go around telling people that ‘just look at him*,* he has drop;’ then find yourself being laughed at by everyone (male*,* 26*,* IDI*,* DREAMS).*

Peers were described as people with similar experiences and to whom it is was easy to talk and thereby support the young person explore their candidacy. However, limitations in their expertise were also acknowledged – both in terms of a lack of knowledge and their lack of professionalism leading to a possible breaching of confidentiality.

## Discussion

Health communication for youth is an important part of ensuring their sexual and reproductive health. There are however barriers to youth becoming aware that they need information and being able to access it. In this paper we examined youths’ candidacy for SRH information by examining how such SRH communication is perceived and received. We found that youths’ identification of their candidacy for SRH information was affected by their fear of infection (either through witnessing illness or hearing stories of infection’s consequences), realisation of a lack of knowledge and concern about personal signs and symptoms. However, the perceptions of some authority figures (e.g., parents, health care providers) whom youths ask for SRH information affected the latter’s willingness to approach the former. This hesitancy was sometimes reinforced by the advice given being to practice sexual abstinence using fear-based messages with warnings of dire consequences of unintended pregnancies and HIV infection.

While youth usually had physical proximity to SRH information sources, actual access was limited by not perceiving parents and other older family as candidates to provide such information, a pattern seen elsewhere in South Africa [50, 51] and beyond [52]. Physical barriers (like distance to clinic and long waiting times) were mentioned as reasons for not accessing services at health centres, but the permeability of such services was also affected by the reception youth seeking help received from clinic staff [53]. Being questioned as to why they as an unmarried adolescent needed contraception or an HIV or pregnancy test, communicated to youth that those services were not intended for them [54]. Such treatment by clinic staff extends beyond clinically trained personnel: a security guard or cleaner can be an important service gatekeeper [55]. Appearing for a service – especially an informational one – and “asserting candidacy” is unlikely to happen in such circumstances, unless the need is acute (e.g., pregnancy complications or an AIDS-related illness).

It is not surprising, therefore, that friends and same-sex siblings were often more comfortable and comforting sources of information, as seen elsewhere in Africa for both women [56] and men [57, 58]. These peers also provided a route to other sources of information, by citing their own experience of using a particular service or by reassuring youth that seeking information from more authoritative figures would not lead to negative consequences. While not true in all cases, for some youth parents played a similar role, linked to their own experience as an adolescent and a desire to be open with, and supportive of, their children.

Youths navigated their way across information boundaries, seeking to control what they shared and with whom, to gain SRH information as painlessly as possible. Teachers and healthcare providers were seen to provide more biological and factual information, on issues such as HIV, STIs, PrEP, circumcision and condoms, but were not sought for support on emotional aspects of sexuality [59, 60]. Central to this analysis, the barriers to youth asserting candidacy for communication with this group include both negative perceptions of the reaction on youth’s presentation for information, and the absence of a perception from healthcare providers that youth should be candidates for SRH advice.

In contrast, peers and same-sex siblings provided emotional support relating to SRH, allow sharing of secrets, experiences and dating advice, but biomedical information from this group could be misleading, inaccurate and incomplete. Where peers can be equipped with accurate information and an ability to facilitate access to a service peer-led SRH communication can be effective [61, 62]. However, changing youth peer social environments to become SRH-promoting requires substantial and appropriate resource inputs to create an atmosphere in which helpful advice can be shared [63].

One risk of the multiple sources of SRH available to youth was that information was conflicting, as well as being incorrect, as seen with the circumcision discussion above. There was insufficient data on this topic in the data analyzed to provide systematic insight into this issue, but focused investigation could allow specific information sources to be targeted as change agents where their expertise is particularly trusted.

### Strengths and limitations

This study had the strength of being able to draw on data collected from multiple perspectives and with multiple aims yet covering topics related to SRH for youth, allowing us to carefully triangulate the data and our findings. However, this secondary data analysis approach, with none of the studies focusing exclusively on the topic of this paper, limited the depth of data on the specific research questions we sought to answer, meaning that further focused research and analysis of these topics in newly collected data could be useful in confirming our initial findings. As ever, the geographical limits of the data collection mean that care needs to be taken in generalizing findings elsewhere, but the social and economic context described is common in many rural and lower-income settings, making it likely that several aspects of our findings are more widely applicable.

## Conclusions

Youth in this area of rural South Africa become aware that they are candidates for SRH information largely due to ad hoc experiences rather than through a systematic sensitization from family, professionals or institutions. Once aware of the need for information, they were able to identify multiple potential sources and often decide which source was most appropriate. However, formal information providers frequently created barriers to access for positive SRH information due to their preconceptions about what activities were appropriate for youth to be doing. In contrast, peers were more open, supportive and provided experiential information, but were perceived to lack trustworthy factual information. Both aspects suggest the potential for formal and informal providers of SRH information and interventions to improve youth SRH knowledge and practice.

## Data Availability

The data underlying this research are not publicly available given the potentially identifiable nature of much of the material.

## List of abbreviations

AGYW: Adolescent girls and young women
AHRI: Africa Health Research Institute
ART: Antiretroviral Therapy
DREAMS: Determined, Resilient, Empowered, AIDS-free, Mentored and Safe
FGD: Focus group discussion
GBV: Gender-based violence
HIV: Human immunodeficiency virus
IDI: In-depth interview
KZN: KwaZulu-Natal
PIP: Population intervention platform
PrEP: Pre-exposure prophylaxis
REC: Research ethics committee
SRH: Sexual and reproductive health
STI: sexually transmitted infection
UKZN: University of KwaZulu-Natal
